# How the nursing work environment moderates the relationship between clinical judgment and person-centered care among intensive care unit nurses

**DOI:** 10.1371/journal.pone.0316654

**Published:** 2025-01-03

**Authors:** Mi Hwa Seo, Eun A. Kim, Hae Ran Kim

**Affiliations:** 1 Department of Nursing, Chonnam National University Hwasun Hospital, Gwangju, Republic of Korea; 2 Department of Nursing, Honam University, Gwangju, Republic of Korea; 3 Department of Nursing, College of Medicine, Chosun University, Gwangju, Republic of Korea; Jordan University of Science and Technology, JORDAN

## Abstract

**Background:**

Person-centered care focuses on individualized care that respects patients’ values, preferences, and autonomy. To enhance the quality of critical care nursing, institutions need to identify the factors influencing ICU nurses’ ability to provide person-centered care. This study explored the relationship between clinical judgment ability and person-centered care among intensive care unit (ICU) nurses, emphasizing how the ICU nursing work environment moderates this relation.

**Methods:**

A cross-sectional survey was conducted between September 4 and September 18, 2023, with 192 ICU nurses recruited from four general hospitals with a convenience sample (valid response rate = 97.4%). Participants completed online self-report structured questionnaires. The collected data were analyzed using hierarchical multiple regression and PROCESS macro Model 1, with a 95% bias-corrected bootstrap confidence interval to verify moderating effects.

**Results:**

Clinical judgment ability (β = .24, p < .001) and ICU nursing work environment (β = .50 p < .001) were found to be significant predictors of person-centered care. These two predictors explained the 47.0% of person-centered care in the final hierarchical regression model. Additionally, Clinical judgment (B = 0.28, p < .001, Boot. 95%CI = 0.13~0.42) and the ICU nursing work environment (B = 0.41, p < .001, Boot. 95%CI = 0.30~0.52) positively affected person-centered care, and the interaction term of clinical judgment and ICU nursing work environment (B = 0.16, p = .026, Boot. 95%CI = 0.02~0.30) also positively affected person-centered care. The moderating effect was particularly significant when the ICU nursing work environment score was 2.90 points (below 14.6%, above 85.4%) or higher on a scale of 1–5 and As the ICU nursing work environment score increased, the positive moderating effect also increased.

**Conclusions:**

The ICU nurses’ clinical judgment ability positively affected person-centered care, and the nursing work environment moderated the relationship between clinical judgment ability and person-centered care. Therefore, strategies for enhancing person-centered care among ICU nurses should focus on developing educational programs to improve clinical judgment ability and implementing comprehensive efforts to effectively improve and manage the nursing work environment.

## Introduction

The intensive care unit (ICU) is a hospital unit where patients with the patients with the highest acuity are admitted and receive intensive monitoring and treatment to sustain life [1]. The emphasis on patient participation in the treatment process by the World Health Organization (WHO) suggests that patient autonomy and active involvement in decision-making are becoming increasingly important [2]. However, the ICU environment often prioritizes urgent life-sustaining tasks over individualized care tailored to the patient’s needs [3]. Additionally, advanced equipment essential for life support can impede nurse–patient interaction, making it difficult for patients to maintain self-esteem owing to their high dependency [4]. Consequently, ICU patients may experience anxiety and isolation owing to their disconnection from family and external support systems [5]. Thus, there is a growing demand to shift the ICU nursing paradigm from survival- to person-centered care that considers individual uniqueness, necessitating research efforts to improve overall treatment outcomes through nursing practices that respect patient individuality [6].

Person-centered care focuses on addressing the individual needs of the patient, respecting their choices and ethical considerations, and protecting their autonomy and dignity [7]. Person-centered care improves the quality of nursing services, enhances patient and family satisfaction, increases nurse job satisfaction, shortens hospital stays, improves patient outcomes, reduces medical costs, and promotes the efficient use of hospital resources [8, 9]. These earlier findings underscore the importance of integrating person-centered care in ICU nursing practice [10]. In the ICU, person-centered care can be implemented in various clinical situations. For example, nurses use non-verbal communication methods for an intubated patient to explain the patient’s condition and maintain autonomy through eye contact or simple gestures [3, 5]. Nurses can talk to the patient about everyday topics (e.g., news, hobbies, interests) [4]. Adjust lighting according to the patient’s preference at night [10]. Even when the patient is sedated or receiving high doses of narcotics, nurses take steps to maintain the patient’s dignity by performing skin care, changing positions, and ensuring family connections to promote comfort and respect [3, 4, 6]. However, ICU nurses often face challenges in implementing person-centered care. This is frequently owing to relationships with other health care professionals, communication difficulties with patients and families, high knowledge and skills required, and heavy workload. These factors contribute to higher stress levels than other departments [11]. To enhance the quality of critical care nursing, institutions need to identify the factors influencing ICU nurses’ ability to provide person-centered care.

Professional competencies—self-awareness, clarity of values and beliefs, commitment to duties, interpersonal skills, knowledge, skills, and judgment—are fundamental prerequisites for performing person-centered care [12]. Notably, nurses’ professional competencies significantly influence the delivery of person-centered care [13]. Among these competencies, clinical judgment is a critical core competency for ICU nurses, encompassing the interpretation or conclusion about patient needs, concerns, or health problems, decisions to act, use or modification of standard guidelines, and decisions to improve approaches based on patient responses [14]. Clinical judgment involves clinical reasoning, decision-making, and critical thinking and is integral across various sub-competencies required for person-centered ICU nursing [15]. Clinical judgment is achieved by interpreting and integrating clinical data into the nursing process, leading to optimal outcomes in complex and urgent medical environments [16]. It is a factor that promotes patient safety management and positive health outcomes [9, 17]. Therefore, clinical judgment can be inferred as a key factor in ICU nurses’ performance of person-centered care. Recognizing nurses’ critical role and importance in providing person-centered care highlights the need for more extensive research focused on this group [1]. Nurses are directly involved in patient care and provide valuable insights into the nursing environment and the practical aspects of person-centered care. However, there is a lack of empirical studies in Korea examining the relationship between clinical judgment and person-centered care among ICU nurses, with existing research primarily focusing on the effects of simulation education for nursing students to enhance clinical judgment in critical care [9, 10, 15, 18]. Thus, our study aimed to identify the level of clinical judgment among ICU nurses and examine its impact on person-centered care.

The nursing work environment supports nurses in providing professional patient care, encompassing the physical environment perceived by nurses and the organizational and policy aspects that influence interactions and work within the hospital [19]. The nursing work environment is a prerequisite and essential factor for delivering high-quality person-centered care [20]. It is a significant predictor of person-centered care performance—a positively perceived work environment can facilitate the delivery of person-centered care [21]. The organizational work culture influences nurses’ performance of person-centered care—adequate staffing and physical support are necessary for improving the nursing work environment [22].

However, research has also suggested that the nursing work environment does not influence the performance of person-centered care [10] nor demonstrate a moderating effect on the relation between nursing competence and person-centered care performance [13]. When the relation between independent and dependent variables is inconsistent across studies, or when personal characteristics or situational factors may affect the strength or direction of this relation, then the moderating effects need to be examined [23]. Studies have suggested that clinical judgment, a core competency in critical care nursing, and the ICU nursing work environment positively impact person-centered care. However, research is significantly lacking on how the nursing work environment’s level modifies this relation’s strength or direction.

**Fig 1:**
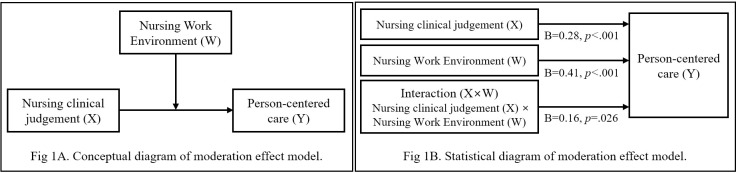
Moderation effect of nursing work environment.

Therefore, this study aimed to identify the factors influencing ICU nurses’ performance of person-centered care, focusing on clinical judgment and the ICU nursing work environment. Particular attention was given to the moderating effect of the ICU nursing work environment (W, moderating variable) on the relationship between clinical judgment (X, independent variable) and person-centered care (Y, dependent variable) (Fig 1). This study sought to provide foundational data for developing nursing human resources, improving the nursing work environment, and establishing effective management strategies to enhance ICU nurses’ performance of person-centered care.

## Materials and methods

### Study design and participants

This study adopted a descriptive survey method. We conveniently sampled participants from four hospitals located in three regions (G City, G Province, and J Province). These hospitals were selected to ensure a diverse representation of ICU nurses, contributing to the variability of the study sample. We included nurses who had been working for more than three months and were directly involved in patient care in the ICU. The participants in this study were selected from nurses who voluntarily agreed in writing to participate after understanding the purpose of the study. Nurses with less than three months of experience were excluded owing to their adjustment and orientation period, which could affect their perception of the nursing work environment and clinical judgment [24]. Additionally, we excluded nurse managers because the ICU nursing work environment measurement tool includes items evaluating leadership.

Using G*Power 3.1.9.4, we calculated the minimum sample size required for multiple regression analysis with a significance level (α) of .05, power of 90%, and effect size of 0.13 [10, 13, 24], and 12 variables (two variables and 10 characteristics). The minimum sample size was 179. Considering a dropout rate of approximately 10%, we collected data from 197 participants. After excluding five incomplete-response participants, we included data from 192 participants (valid response rate = 97.4%) in the final analysis. The final research results showed that the effect size according to the regression analysis result was 0.89 (using G*Power 3.1.9.4), and the conditional effect was 0.17–0.40 (Table 5). Therefore, this study’s effect size estimate (0.13) was appropriate.

### Ethical approval

The study was conducted per the Declaration of Helsinki and was approved by the Institutional Review Board of Chonnam National University Hwasun Hospital (Approval No: CNUHH-2023-169). We explained the purpose of the study to the responsible parties at each institution and obtained consent, followed by the announcement of participant recruitment. The responsible parties provided details about the study to potential participants, and the researchers further explained the study’s purpose to those who wished to participate and obtained their consent. The data collection period was between September 4 and September 18, 2023. The online survey took an average of 15 min, and participants received a gift voucher (3.71$) as compensation for their time. The consent form included information on the benefits and risks of participation, confidentiality and privacy guarantees, voluntary consent, the possibility of withdrawal without any disadvantage, use of data solely for research purposes, and automatic destruction of data three years after the study’s completion.

### Study instrument

#### Clinical judgment

We measured clinical judgment using the Nursing Clinical Judgment Scale, developed and validated in Korea [25]. The tool consists of 23 items across six sub-factors: integrated data analysis (six items), intervention evaluation and reflection (three items), intervention rationale (four items), professional consultation (three items), patient-centered care (four items), and collaboration with fellow nurses (three items). Each item is rated on a five-point Likert scale from 1 (not at all) to 5 (very much), with higher scores indicating higher clinical judgment. The tool’s reliability (Cronbach’s α) at the time of development was .92 overall, with sub-factor reliabilities ranging from .74 to .85 [30]. Our study’s overall reliability was .93, with sub-factor reliabilities ranging from .77 to .88.

#### ICU nursing work environment

We assessed the ICU nursing work environment using the Korean Nursing Work Environment Scale for Critical Care Nurses [26]. The tool consists of 21 items across four sub-factors: authentic leadership (eight items), organizational culture (six items), adequate staffing (four items), and professional practice (three items). Each item is rated on a five-point Likert scale from 1 (not at all) to 5 (very much), with higher scores indicating a more positive perception of the ICU nursing work environment. The tool’s reliability (Cronbach’s α) at the time of development was .92 overall, with sub-factor reliabilities ranging from .67 to .88 [24]. In our study, the overall reliability was .95, with sub-factor reliabilities ranging from .73 to .96. We consider sub-factor reliabilities above .70 acceptable, thus ensuring the internal consistency of our scale.

#### Person-centered care in the ICU

Using the Person-centered Critical Care Nursing tool developed and validated in Korea, we measured person-centered care in the ICU [27]. The tool consists of 15 items across four sub-factors: empathy (four items), individuality (four items), respect (four items), and comfort (three items). Each item is rated on a five-point Likert scale from 1 (not at all) to 5 (very much), with higher scores indicating higher performance of person-centered care by ICU nurses. The tool’s reliability (Cronbach’s α) at the time of development was .84 overall, with sub-factor reliabilities ranging from .71 to .81 [31]. Our study’s overall reliability was .86, with sub-factor reliabilities ranging from .72 to .76.

#### Statistical analysis

The collected data were analyzed using SPSS/WIN 28.0 (IBM Corp., Armonk, NY, USA) and SPSS PROCESS macro (version 4.2) [28]. The specific methods were as follows:

We used descriptive statistics, including frequency, percentage, mean, and standard deviation, to analyze the characteristics of the participants, clinical judgment, ICU nursing work environment, and person-centered care.

We analyzed differences in clinical judgment, ICU nursing work environment, and person-centered care according to participants’ characteristics using independent *t*-tests and one-way ANOVA after testing for normality (skewness 1. < |3|, kurtosis < |10|) [29]. Post-hoc tests were conducted using Scheffé’s test. If the assumption of homogeneity of variance was not met, Welch’s and Games-Howell’s post-hoc tests were used.

We analyzed the relations among clinical judgment, ICU nursing work environment, and person-centered care using Pearson’s correlation coefficients (bivariate correlation coefficient).

Using hierarchical multiple regression, we analyzed the factors influencing person-centered care performance among ICU nurses.

We analyzed the moderating effect of the ICU nursing work environment on the relation between clinical judgment and person-centered care using PROCESS macro Model 1 (bootstrapping 5,000 times, 95% confidence interval). We determined the significance of the moderating effect by the importance of the regression coefficient of the interaction term (p < .05) and the absence of 0 in the 95% confidence interval. After testing the moderating effect, we examined the conditional effects at specific values of the moderator using simple slope analysis (pick-a-point method) and Johnson-Neyman technique [28, 30].

## Results

### General characteristics

Table 1 presents the general characteristics of the 192 participants. Of the total respondents, most (90.6%) were women, reflecting the gender distribution in nursing. The average age of the participants was 32.78 years, with a significant portion being unmarried (60.4%). Most participants held a bachelor’s degree (87.0%), indicating a well-educated workforce. Regarding hospital types, 79.2% of the participants worked in tertiary hospitals, whereas 20.8% were in general hospitals. In terms of ICU settings, the distribution included medical ICUs (51.0%), surgical ICUs (27.6%), and emergency ICUs (21.4%). The total work experience was 8.98 years, with an average ICU work experience of 2.95 years. Notably, most participants (73.4%) were familiar with person-centered care, but only a small proportion (18.2%) had received relevant education.

**Table 1:** Differences in clinical judgment ability, intensive care unit nursing work environment, and person-centered care according to participant’s characteristics (N = 192).

Characteristics	Categories	n(%)	Clinical judgment ability		ICU nursing work environment		Person-centered care	
			M ± SD	t or F (*p*) post-hoc^†^	M ± SD	t or F (*p*) post-hoc^†^	M ± SD	t or F (*p*) post-hoc^†^
Sex	Men	18(9.4)	3.58 ± 0.49	**-**2.83 (.005)	3.07 ± 0.73	-3.39 (< .001)	3.30 ± 0.62	-1.88 (.062)
	Women	174(90.6)	3.91 ± 0.48		3.60 ± 0.63		3.55 ± 0.54	
Age (years) (M±SD: 32.78±6.85)	≦29	83(43.2)	3.85 ± 0.52	0.42 (.661)	3.55 ± 0.69	0.62^‡^ (.540)	3.50 ± 0.59	0.83 (.437)
	30–39	71(37.0)	3.92 ± 0.49		3.61 ± 0.71		3.59 ± 0.52	
	≥40	38(19.8)	3.90 ± 0.40		3.46 ± 0.45		3.46 ± 0.50	
Marital status	Single	116(60.4)	3.83 ± 0.50	-1.72 (.087)	3.51 ± 0.70	-1.20 (.230)	3.46 ± 0.55	-1.96 (.051)
	Married	76(39.6)	3.96 ± 0.46		3.62 ± 0.57		3.62 ± 0.53	
Education	Associate	6(3.1)	4.19 ± 0.38	2.27 (.106)	3.70 ± 0.50	0.31 (.735)	3.87 ± 0.49	1.23 (.295)
	Bachelor	167(87.0)	3.86 ± 0.50		3.56 ± 0.68		3.52 ± 0.56	
	≥ Master	19(9.9)	4.03 ± 0.36		3.47 ± 0.40		3.49 ± 0.46	
Hospital type	Tertiary	152(79.2)	3.88 ± 0.49	-0.27 (.785)	3.61 ± 0.63	2.41 (.017)	3.56 ± 0.53	1.62 (.108)
	General	40(20.8)	3.90 ± 0.49		3.33 ± 0.70		3.40 ± 0.59	
Type of ICU*	Medical	98(51.0)	3.95 ± 0.45	1.74 (.178)	3.72 ± 0.62	6.64 (.002) a>b,c	3.63 ± 0.58	5.21^‡^ (.006) a>b
	Surgical	53(27.6)	3.84 ± 0.55		3.35 ± 0.67		3.34 ± 0.52	
	Emergency	41(21.4)	3.80 ± 0.48		3.42 ± 0.64		3.52 ± 0.43	
Total career (years) (M±SD: 8.98±7.15)	0.33–<5	73(38.0)	3.86 ± 0.58	0.69^‡^ (.503)	3.55 ± 0.74	0.21^‡^ (.815)	3.53 ± 0.57	0.01 (.995)
	5–<10	59(30.7)	3.85 ± 0.42		3.51 ± 0.67		3.52 ± 0.54	
	≥10	60(31.3)	3.94 ± 0.42		3.59 ± 0.52		3.52 ± 0.54	
ICU career (years) (M±SD: 2.95±2.33)	0.33–<1	26(13.5)	3.95 ± 0.58	0.60 (.615)	3.78 ± 0.80	1.57 (.198)	3.61 ± 0.57	0.40 (.753)
	1–<3	89(46.4)	3.87 ± 0.48		3.56 ± 0.60		3.50 ± 0.55	
	3–<5	42(21.9)	3.83 ± 0.51		3.52 ± 0.66		3.56 ± 0.48	
	≥5	35(18.2)	3.95 ± 0.41		3.42 ± 0.65		3.48 ± 0.61	
Have you ever heard about person-centered care	Yes	141(73.4)	3.91 ± 0.48	1.41 (.161)	3.57 ± 0.67	0.52 (.605)	3.56 ± 0.56	1.28 (.203)
	No	51(26.6)	3.80 ± 0.50		3.51 ± 0.62		3.44 ± 0.50	
Education experience of person-centered care	Yes	35(18.2)	3.94 ± 0.45	0.75 (.455)	3.61 ± 0.59	0.52 (.603)	3.63 ± 0.55	1.25 (.212)
	No	157(81.8)	3.87 ± 0.49		3.54 ± 0.67		3.50 ± 0.55	

M: Mean, SD: Standard deviation, ICU: intensive care unit.

^†^F = analysis of variance, Post-hoc: Scheffé test.

^‡^F = Welch analysis of variance, post-hoc: Games–Howell test.

### Clinical judgment, intensive care unit nursing work environment, and person-centered care

Table 2 shows the scores for the research variables. The scores for each variable were based on a total of five points. The mean scores were clinical judgment 3.88, nursing work environment 3.55, and person-centered care 3.53. The normality of each variable was confirmed by skewness (absolute value < 3) and kurtosis (absolute value < 10) [29], indicating that all variables followed a normal distribution.

**Table 2:** Degree of clinical judgment ability, intensive care unit nursing work environment, and person-centered care (N = 192).

Variables/Sub-categories	Possible range	Item range	Skewness	Kurtosis	M ± SD
Clinical judgment ability	1–5	2.35–5.00	-0.14	0.27	3.88 ± 0.49
Integrated data analysis	1–5	1.67–5.00	-0.27	0.93	3.79 ± 0.61
Evaluation and reflection on interventions	1–5	1.67–5.00	-0.48	0.54	3.84 ± 0.67
Evidence on interventions	1–5	2.25–5.00	-0.40	0.34	4.09 ± 0.57
Collaboration with health professionals	1–5	1.00–5.00	-0.64	0.97	3.77 ± 0.80
Patient-centered nursing	1–5	2.00–5.00	-0.20	-0.19	3.68 ± 0.65
Collaboration between colleague nurses	1–5	2.00–5.00	-0.69	0.91	4.23 ± 0.62
Nursing work environment	1–5	1.33–5.00	-0.23	0.69	3.55 ± 0.65
Authentic leadership	1–5	1.13–5.00	-0.71	1.23	3.86 ± 0.75
Organizational culture	1–5	1.00–5.00	-0.41	0.70	3.59 ± 0.73
Staffing adequacy	1–5	1.00–5.00	0.00	-0.25	2.93 ± 0.91
Professional practice	1–5	1.00–5.00	-0.19	0.49	3.50 ± 0.71
Person-centered care	1–5	1.47–4.87	-0.07	0.33	3.53 ± 0.55
Compassion	1–5	1.75–5.00	-0.21	-0.13	3.73 ± 0.68
Individuality	1–5	1.00–5.00	-0.10	-0.44	2.90 ± 0.84
Respect	1–5	1.00–5.00	-0.37	0.49	3.66 ± 0.70
Comfort	1–5	2.00–5.00	-0.37	0.23	3.91 ± 0.64

M: Mean, SD: Standard deviation.

### Differences in clinical judgment, intensive care unit nursing work environment, and person-centered care by participant characteristics

The clinical judgment mean score of ICU nurses showed statistically significant differences by sex (Table 1), with female nurses (3.91) scoring significantly higher than male nurses (3.58). Perceptions mean score of the ICU nursing work environment also showed significant differences by sex, type of hospital, and ICU. Female nurses (3.60) had more positive perceptions than male nurses (3.07); nurses in tertiary hospitals (3.61) had more positive perceptions than those in general hospitals (3.33), with post hoc tests indicating that nurses in medical ICU (3.72) had significantly more positive perceptions than those in emergency (3.42) or surgical (3.35) ICU. Differences in person-centered care mean score by the type of current ICU were also significant, with post hoc tests indicating that nurses in medical ICUs (3.63) scored significantly higher than those in surgical ICUs (3.34).

### Correlations between clinical judgment, intensive care unit nursing work environment, and person-centered care

As shown in Table 3, person-centered care among ICU nurses was significantly positively correlated with clinical judgment (r = .59, p < .001) and the ICU nursing work environment (r = .66, p < .001). Clinical judgment was also significantly positively correlated with the ICU nursing work environment (r = .54, p < .001).

**Table 3:** Correlation among variables (N = 192).

Variables	Clinical judgment ability r (*p*)	Intensive care unit nursing work environment r (*p*)
Intensive care unit nursing work environment	.54 (< .001)	
Person-centered care	.59 (< .001)	.66 (< .001)

### Impact of clinical judgment and intensive care unit nursing work environment on person-centered care

We conducted a hierarchical multiple regression analysis to identify factors influencing person-centered care among ICU nurses. Variables that showed significant univariate correlations with person-centered care (clinical judgment, ICU nursing work environment) and general characteristics that significantly differed with person-centered care (type of current ICU) were included as dummy variables in the analysis. Before regression analysis, we performed basic assumption tests for multicollinearity. As shown in Table 4, the tolerance was greater than .10, and the variance inflation factor was less than 10, indicating no multicollinearity. The Durbin–Watson statistic was close to 2, suggesting no autocorrelation of errors. We confirmed the normality and homoscedasticity of residuals through histograms, normal P-P plots, and scatter plots, which indicated that all assumptions were satisfied. Cook’s distance values were less than 1.0, indicating no outliers, and the regression model was deemed appropriate.

**Table 4:** Predictors of person-centered care (N = 192).

Variables	Category	Model 1					Model 2				
		B	SE	β	t	p	B	SE	β	t	p
	(Constant)	1.28	0.27		4.77	< .001	1.06	0.24		4.45	< .001
Type of ICU*^†^	Medical	0.03	0.09	.03	0.33	.743	-0.05	0.08	-.04	-0.62	.538
	Surgical	-0.20	0.10	-.17	-2.14	.034	-0.16	0.08	-.13	-1.91	.057
Clinical judgment ability		0.59	0.07	.52	8.62	< .001	0.27	0.07	.24	3.61	< .001
ICU Nursing work environment							0.42	0.06	.50	7.50	< .001
R^2^		.32					.48				
Adjusted R^2^		.31					.47				
ΔR^2^ (*p*)							.16 (< .001)				
F (*p*)		29.61 (< .001)					42.79 (< .001)				

B: Unstandardized coefficient; β: Standardized coefficient; SE: Standard error.

*ICU = intensive care unit. ^†^Reference group = Emergency.

Dubin–Watson = 1.84; Tolerance = .58 to .98; Variance inflation factor = 1.02 to 1.71

Table 4 also shows that in Model 1, which included clinical judgment and type of current ICU, clinical judgment (positive) and working in a surgical ICU (negative) were significant factors influencing person-centered care among ICU nurses. Model 1 explained 31.0% of the variance. In Model 2, which added the ICU nursing work environment, both the ICU nursing work environment and clinical judgment were significant positive factors influencing person-centered care. Based on its adjusted R^2^, Model 2 explained 47.0% of the variance, a substantial increase of 16% from Model 1. A positive post-hoc value indicates that higher clinical judgment is associated with greater person-centered care. In contrast, a negative post-hoc value for surgical ICUs suggests that these settings are linked to lower levels of person-centered care than other ICUs.

### Moderating effect of intensive care unit nursing work environment on the relation between clinical judgment and person-centered care

To investigate the moderating effect of the ICU nursing work environment on the relation between clinical judgment (independent variable, X) and person-centered care (dependent variable, Y), we used PROCESS macro Model 1. The type of current ICU was controlled as a dummy variable. The results, presented in Table 5, showed that clinical judgment (X) and the ICU nursing work environment (W) significantly affected person-centered care (Y), and the interaction term of clinical judgment (X) and ICU nursing work environment (W) also positively affected person-centered care (Y). All effects were significant, with 95% confidence intervals excluding zero, confirming the moderating impact of the ICU nursing work environment (Fig 1).

**Table 5:** Moderating effect of intensive care unit nursing work environment on the relation between clinical judgment ability and person-centered care (N = 192).

Moderation model		B	SE	t	p	Boot. 95%CI	
						LLCI	ULCI
(Constant)		3.57	0.06	56.28	< .001	3.44	3.69
Clinical judgment ability (X → Y)		0.28	0.07	3.77	< .001	0.13	0.42
Intensive care unit nursing work environment (W → Y)		0.41	0.06	7.39	< .001	0.30	0.52
Interaction: Clinical judgment ability × intensive care unit nursing work environment (X×W → Y)		0.16	0.07	2.24	.026	0.02	0.30
R^2^		.49					
ΔR^2^ (*p*)		.02 (< .001)					
F (*p*)		35.97, < .001					
Conditional direct effect	**ICU Nursing work environment**	**Effect**	**SE**	**t**	**p**	**Boot. 95%CI**	
						**LLCI**	**ULCI**
	Mean-1SD (2.90)	0.17	0.08	2.04	.043	0.01	0.34
	Mean (3.55)	0.28	0.07	3.77	< .001	0.13	0.42
	Mean+1SD (4.21)	0.40	0.09	4.28	< .001	0.20	0.55

Johnson–Neyman significance region = 2.90 (below 14.6%, above 85.4%)

X: Independent variable (clinical judgment ability). Y: Dependent variable (person-centered care), W: Moderating variable (intensive care unit nursing work environment).

B: Unstandardized coefficient, SE: Standard error, Boot.: Bootstrapping, CI: confidence interval, LLCI: Lower limit confidence interval, ULCL: Upper limit confidence interval, SD: Standard deviation.

Conditional effects analysis through simple slope analysis showed that the relation between clinical judgment and person-centered care was significant at all ICU nursing work environment levels. Table 5 also shows the mean values below, at, and above. The results indicated that person-centered care varied with the ICU nursing work environment level, confirming the moderating effect.

**Fig 2:**
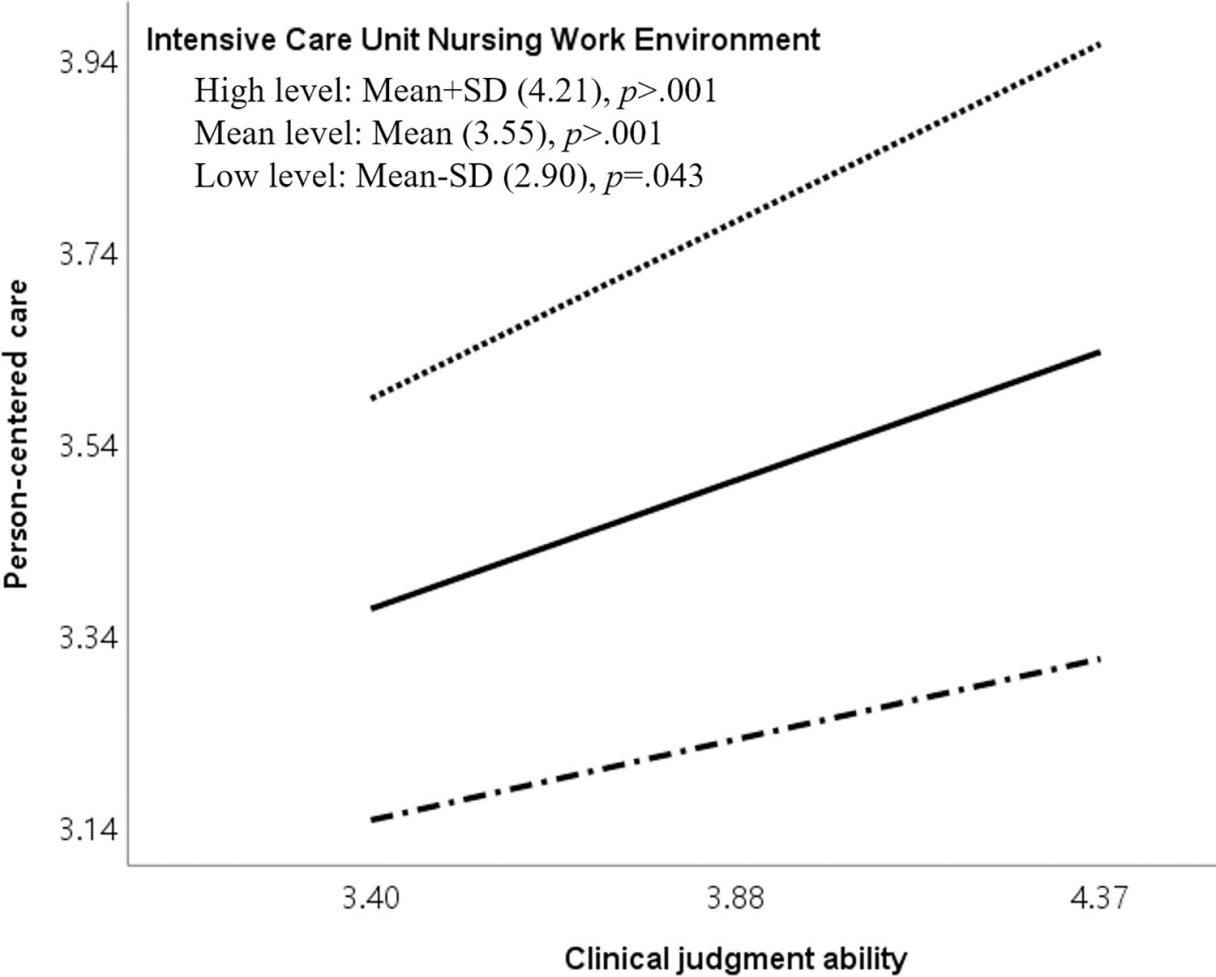
Visual representation of the moderation effect of intensive care unit nursing work environment on clinical judgment ability and person-centered care.

The Johnson–Neyman analysis revealed that the moderating effect of the ICU nursing work environment was significant when the score was 2.90 or above (possible range: 1–5), encompassing 85.4% of participants. As the ICU nursing work environment score increased, the positive moderating effect also increased. Thus, the impact of clinical judgment on person-centered care strengthened as the ICU nursing work environment improved (Table 5, Fig 2).

## Discussion

This study attempted to investigate the impact of clinical judgment ability and ICU nursing work environment on person-centered care among ICU nurses in South Korea, aiming to elucidate the moderating effect of the ICU nursing work environment on the relation between clinical judgment ability and person-centered care.

This study rated person-centered care among ICU nurses at 3.53 points. This result is similar to previous research in Korea using the same instrument [3]. Additionally, we noted differences in person-centered care scores depending on the type of ICU—nurses in medical ICUs had higher scores than those in surgical ICUs. This difference may be attributed to the nature of ICUs. In medical ICUs, practicing person-centered care is perceived to be of greater importance owing to the need for continuous and long-term patient management. Conversely, in surgical ICUs where acute patient management is predominant, person-centered care may be less emphasized [31].

Our study also indicated that medical ICUs were evaluated more positively regarding the nursing work environment than surgical ICUs. A possible reason is that medical ICUs with higher proportions of long-term hospitalized patients create a conducive environment for patient-centered care. This can enhance nurses’ job satisfaction by allowing them to perform their duties more effectively [32]. Therefore, optimizing the work environment tailored to the characteristics of ICUs is essential. Institutions should support conditions where nurses can provide patient-centered care and enhance teamwork to improve job satisfaction. We also found that male nurses perceived the nursing work environment less positively than female nurses. Although the number of male nurses is increasing in the nursing profession, they often experience discrimination in areas such as departmental assignments owing to gender stereotypes and social barriers [33]. Nursing managers should understand and address the satisfaction and needs of male nurses in their work environment.

Our findings align with previous research [3, 15, 20], confirming a significant positive correlation among clinical judgment ability person-centered care among ICU nurses, and the ICU nursing work environment. This indicates that the work environment in ICUs plays a crucial role in influencing nurses’ clinical judgment abilities and their practice of person-centered care. Strategies for improving nursing practice in ICUs and enhancing nurses’ professional capabilities can be guided by a thorough understanding of the environmental conditions within ICUs.

This study aimed to elucidate how the ICU nursing work environment moderates the relation between clinical judgment ability and person-centered care among ICU nurses, highlighting the significant implications of the ICU nursing work environment on person-centered care. The findings indicated that in groups with higher ICU nursing work environment scores, nurses with more vital clinical judgment abilities tended to exhibit higher levels of person-centered care. This suggests that when ICU nurses with excellent clinical judgment abilities promote person-centered care, their working environment further enhances these positive effects. The Johnson–Neyman analysis underscored the critical nature of the moderating impact of the ICU nursing work environment, particularly noting significant effects when scores were above 2.90 points. When ICU nursing work environment scores fell below 2.90 points, nurses with lower clinical judgment abilities tended to demonstrate lower levels of person-centered care. Therefore, efforts to enhance person-centered care among ICU nurses are essential through strengthening nurses’ clinical judgment abilities and executing organizational improvements in the work environment.

This study confirmed empirically that the ICU nursing work environment and clinical judgment ability of ICU nurses are significant factors that can enhance person-centered care. The ICU nursing work environment was shown to moderate the relation between ICU nurses’ clinical judgment ability and person-centered care, exerting a positive influence. Specifically, the ICU nursing work environment played a role in positively reinforcing the relation between clinical judgment ability and person-centered care. It is interpreted as a crucial intervention strategy because organizational strategies and improvements aimed at positively transforming the ICU nursing work environment can effectively enhance person-centered care, especially for nurses with lower clinical judgment abilities. This suggests that both the clinical judgment ability and the nursing work environment of intensive care unit nurses play a pivotal role in person-centered care. Practical strategies for improving the ICU nursing work environment include ensuring adequate staffing, promoting leadership that fosters open communication, and providing nurses with the necessary resources to manage patient care efficiently. These findings offer valuable guidelines for improving nursing practices in ICUs and enhancing nurses’ professional capabilities. In particular, improving the ICU nursing work environment while simultaneously strengthening nurses’ clinical judgment abilities through education can be a highly effective strategy for significantly enhancing the quality of person-centered care. Therefore, this study underscores the importance of continuous efforts in developing nursing human resources through enhancing clinical judgment abilities, improving ICU nursing work environments, and establishing efficient management strategies to enhance ICU nurses’ performance of person-centered care.

### Limitations of the study

The study’s main limitation is that it targeted ICU nurses from four hospitals through convenience sampling, making it difficult to generalize the results to all ICU nurses and may not be representative of the entire country. Additionally, the ICU nurses from these hospitals may have diverse backgrounds and experiences, which could influence their perceptions and practices related to person-centered care. This study provides results limited to a specific region, suggesting that it may not reflect the differences in experiences or perceptions among nurses nationwide. Therefore, caution should be exercised when attempting to apply these findings to ICU nurses in other regions or settings, as the specific context of the participating hospitals may not reflect the broader population of ICU nurses.

## Conclusions

This study underscores that clinical judgment ability and ICU nursing work environment are critical factors that can enhance person-centered care. It empirically validates that the ICU nursing work environment moderates the relation between clinical judgment ability and person-centered care, demonstrating its positive influence on care among ICU nurses. It confirms that better ICU nursing work environments can further strengthen the impact of clinical judgment ability on person-centered care. Based on these findings, strategies for enhancing person-centered care among ICU nurses should focus on developing educational programs to improve clinical judgment ability and implementing comprehensive efforts to effectively improve and manage the nursing work environment. Recommendations for future research include developing educational programs to enhance person-centered care performance aligned with strengthened clinical judgment abilities. Additionally, studies are needed to evaluate programs that enhance leadership among nursing unit managers to improve the quality of ICU nursing work environments. Given that this study focused on ICU nurses in specific regions, future studies should expand the scope when conducting repeated research. Furthermore, future research should target ICU nurses from more diverse regions to enhance the generalizability of the findings.

## Supporting information

S1 File Raw data. (XLSX)
